# Effects of Lactoferrin-Fortified Formula on Acute Gastrointestinal Symptoms in Children Aged 12–32 Months: A Randomized, Double-Blind, Placebo-Controlled Trial

**DOI:** 10.3389/fped.2020.00233

**Published:** 2020-05-19

**Authors:** Noriko Motoki, Masaru Mizuki, Teruomi Tsukahara, Momoko Miyakawa, Shutaro Kubo, Hirotsugu Oda, Miyuki Tanaka, Koji Yamauchi, Fumiaki Abe, Tetsuo Nomiyama

**Affiliations:** ^1^Department of Preventive Medicine and Public Health, Shinshu University School of Medicine, Matsumoto, Nagano, Japan; ^2^Food Ingredients and Technology Institute, R&D Division, Morinaga Milk Industry, Co., Ltd., Zama, Kanagawa, Japan

**Keywords:** lactoferrin, infectious disease, diarrhea, growing-up formula, children

## Abstract

**Objective:** We investigated the effects of lactoferrin (LF)-fortified formula on acute gastrointestinal and respiratory symptoms in children.

**Design:** Randomized, double-blind, placebo-controlled trial.

**Setting and subjects:** Children aged 12–32 months in Japan.

**Intervention:** Intake of placebo or LF (48 mg/day)-fortified formula for 13 weeks.

**Primary endpoint:** Prevalence of acute gastrointestinal and respiratory symptom.

**Results:** One hundred nine participants were randomized. Eight participants were lost to follow-up, withdrew consent, or were deemed inappropriate for the trial, with 101 participants receiving complete analyses (placebo group, *n* = 48; LF group, *n* = 53).

**Outcomes:** The prevalence of acute gastrointestinal symptoms was significantly less in the LF group (22/53 [41.5%]) than in the placebo group (30/48 [62.5%], *p* = 0.046). The total number of days having acute respiratory symptoms was significantly lower in the LF group (9.0) than in the placebo group (15.0, *p* = 0.030).

**Harms:** The rate of adverse events was similar between the groups. No adverse drug reactions were found.

**Conclusions:** LF intake decreased the prevalence of acute gastrointestinal symptoms in children aged 12–32 months.

## Introduction

Despite remarkable advances in the improvement of child survival, the mortality rate of children in developing countries remains high. In 2015, 5.9 million children worldwide did not live to the age of 5 years, primarily due to pneumonia (12.8%) and diarrhea (8.6%) (1). In addition to mortality, multiple episodes of and persistent diarrhea may also hinder long-term growth, nutritional uptake, and cognitive development (2).

Breast milk is the ideal nourishment for the healthy growth and development of newborn infants; the World Health Organization (WHO) recommends that mothers breastfeed their children for up to 2 years of age or beyond (3). The advantages of breastfeeding are not restricted to nutritional value and include the transfer of multiple anti-infectious, anti-inflammatory, and immunoregulatory factors such as secretory antibodies, glycans, lactoferrin, leukocytes, and cytokines (4).

Lactoferrin (LF) is an 80 kDa iron-binding mammalian glycoprotein of the transferrin family. It is a component of exocrine secretions such as breast milk and saliva and is present in neutrophil granules (5). LF plays an important role in the immunoregulation and defense against several pathogens (6). The protein is also involved in antimicrobial/antiviral activities, immunomodulatory activity, and antioxidant activity (7). The concentration of LF in breast milk is maintained at high levels even after the child reaches 1 year of age (8).

Breastfeeding is the simplest and most cost-effective intervention for protecting children against diarrhea and all causes of mortality (9). However, there are cases when the mother is unable to breastfeed due to illnesses, medications, insufficient milk production, or a return to the workplace. Infant formula can be given to infants of <1 year of age as a substitute for breast milk when necessary. The formula is fortified with bovine LF in some countries (10) to reportedly suppress diarrhea and respiratory tract infections (11). The rate of breastfeeding for children over 1 year of age is especially low (12) despite the risk of contracting infectious diseases remaining high. Thus, the effects of LF-fortified formulas on children over 1 year of age are also of interest, but have not yet been examined. This randomized, double-blind, placebo-controlled trial investigated the impact of an LF-fortified growing-up formula on acute gastrointestinal and respiratory symptoms in children aged 12–32 months.

## Methods

### Trial Design and Ethical Approval

This was a randomized (1:1), double-blind, placebo-controlled, parallel-group, comparative trial conducted by the Department of Preventive Medicine and Public Health of Shinshu University School of Medicine in Matsumoto, Japan. It was performed in accordance with the current revision of the Declaration of Helsinki (13) and Ethical Guidelines for Medical and Health Research Involving Human Subjects (14). The study's protocol and informed consent form were approved by the Institutional Review Board (IRB) of Shinshu University School of Medicine on October 17, 2017 (approval number: 3847). Written informed consent was obtained from all parents. This investigation was registered at the University Hospital Medical Information Network (UMIN) Clinical Trials Registry in Japan (an international clinical trial registry accepted by the International Committee of Medical Journal Editors) on October 17, 2017 (registration number: UMIN000029685).

### Participants, Eligibility, and Exclusion Criteria

This trial was performed between November 27, 2017 and April 1, 2018. Eligible participants were apparently healthy children between the ages of 12 and 32 months who attended nursery schools in Matsumoto, Japan. Exclusion criteria were allergies to milk or soybeans, receiving breast milk, habitual consumption of LF, history of serious disorders of the liver, kidney, heart, lung, gastrointestinal tract, blood, endocrine system, or metabolic system, or judged to be inappropriate to participate in the trial by the principal investigator.

### Intervention

The participants were randomly allocated into a placebo or LF (48 mg/day) group. The participants' parents were instructed to reconstitute one sachet containing either the placebo or LF-fortified formula with water or boiled water cooled to 50°C and provide their children with one sachet per day during the 13-week intervention period. One sachet contained powdered formula, which was a modified commercial growing-up formula for children aged 12–36 months manufactured by Morinaga Milk Industry, Co., Ltd., Japan. Both the placebo and the LF formulas were composed of the same nutrients except for the addition of LF (48 mg) in the LF formula. LF was replaced by dextrin in the placebo formula. Both test formulas were similar in appearance, texture, smell, and packaging. During the intervention period and 2-week post-intervention period, formula intake (100, 50, or 0%), acute respiratory or gastrointestinal symptoms, any diagnoses made by an examining physician, the names of any medications, and any other physical changes were recorded in diaries by the parents based on previous researches (11, 15).

Diarrhea (once or more loose or liquid stool per day), vomiting, and/or fatigue associated with them were recorded as acute gastrointestinal symptoms. Nasal secretion/congestion and/or cough/sputum, and/or fever (≥38.0°C) and fatigue associated with them were recorded as acute respiratory symptoms. All participants began and completed the administration of the test formula and diary records on the same day. Well-trained public health workers contacted the parents every 4 weeks at nursery schools to verify the intake of the test formulas and diary records, and carefully collected further information regarding symptoms of any acute infections or other issues such as allergic symptoms. Symptoms were confirmed by the principal investigator based on diary described by the parents and information from the public health workers, and ultimately determined to be due to respiratory infections, gastroenteritis, or other issues. If respiratory and gastrointestinal symptoms were considered to be complicated, they were counted in both cases. Vomiting due to coughing was considered a respiratory symptom.

The intake of test formula on each day was recorded as 100, 50, or 0% of one sachet in the diary. Based on these data, the average intake per day during the test period for each participant was calculated. Compliance was expressed as the median of the average intake per day in the placebo or LF group. At the end of the test, the remaining test formulas were collected, and the records were confirmed to be consistent. Parents were instructed in advance so that only the test participants could drink the test formulas.

### Primary and Secondary Endpoints

The primary endpoints were the prevalence of acute gastrointestinal or respiratory symptoms based on diary records described by parents in the interventional period. Prevalence rate was defined as the number of participants who experienced more than a symptom. The secondary endpoints were the total number of days and duration of the symptoms during the interventional period. The total number of days was defined as the cumulative number of symptomatic days and duration was the mean of consecutive days per episode. Individual episodes were separated by at least 1 day.

### Safety Assessment

Any unfavorable or unintended signs, symptoms, or diseases were defined as adverse events. Adverse events were evaluated using the Revised National Cancer Institute - Common Toxicity Criteria Version 4.0, and those events where a causal relationship with the intake of the test formula were identified were designated as adverse drug reactions.

### Sample Size

Children attending nursery schools were reported to have a higher risk of gastrointestinal and respiratory infections than children cared for at home (16). Hatakka et al. reported that the percentage of children with respiratory symptoms reached 95% in 30 weeks (16). Based on an estimated prevalence rate of acute illness of 95%, the ability of LF to reduce the prevalence to 75%, a type 1 error of 0.05, and a power of 80%, we calculated a required sample size of 98 (49 in each group) and set a target sample size of 110 (55 in each group) to account for possible dropouts.

### Randomization, Allocation, and Blinding

An independent allocation manager at a different university produced a computer-generated allocation table using the permuted block method [block size of 4 (1:1 ratio)] according to nursery school. The allocation tables were sealed in an opaque envelope and were kept until key breaking. The investigators, subjects, and public health workers were blind to all information during this period. The investigators enrolled the subjects and assigned them to test formula numbers. Key breaking was performed after locking the database and statistical analysis plan.

### Statistical Analysis

Data are expressed as the mean ± standard deviation or the median (interquartile range). Fisher's exact test was used to analyze the prevalence of symptoms and the Mann-Whitney U test was employed to evaluate the total number of days and duration of any symptoms. Statistical analyses were performed using IBM SPSS Statistics version 24 software (SPSS, Chicago, IL). *P*-values <0.05 were considered statistically significant.

## Results

**Figure 1:**
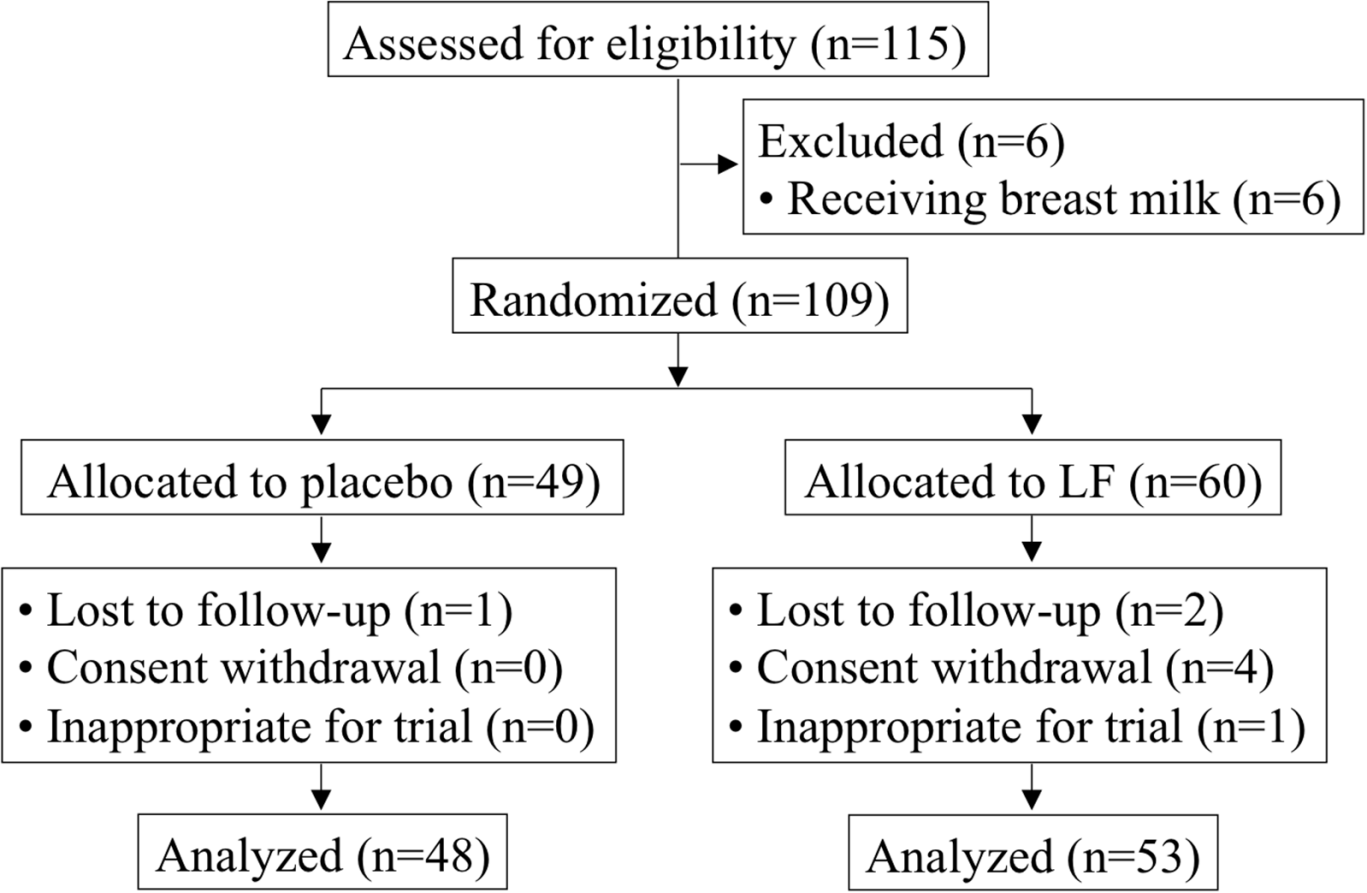
Subject selection flowchart.

Of the 115 participants who provided informed consent, six participants were excluded due to receiving breast milk and 109 participants were enrolled (placebo, *n* = 49; LF, *n* = 60) (Table 1). The mean age was 25.8 ± 4.9 months, and the ratio of boys to girls was 56:53. After randomization, three participants were lost to follow-up with no data available. Four participants withdrew consent. Additionally, one participant was stopped by the principal investigator because of skin eczema and turned out not to satisfy the eligibility. Therefore, eight participants were lost and the resulting full analysis set (FAS) of data (101 participants) were used for the primary analysis (placebo, *n* = 48; LF, *n* = 53) (Figure 1). There were no significant differences between the participants' baseline background in both groups for primary analysis.

**Table 1:** Baseline demographics.

	**Total**	**Placebo**	**LF**
Number	109	49	60
Male, *n* (%)	56 (51.4)	28 (57.1)	28 (46.7)
Age, months	25.8 ± 4.9	25.7 ± 5.2	25.9 ± 4.6
Body height, cm	84.2 ± 5.0	84.2 ± 5.7	84.1 ± 4.4
Body weight, kg	11.5 ± 1.4	11.5 ± 1.5	11.6 ± 1.4
Weight-for-age, z-score	−011. ± 0.98	−0.16 ± 1.00	−0.06 ± 0.98
Height-for-age, z-score	−0.57 ± 1.18	−0.56 ± 1.15	−0.58 ± 1.21
Weight-for-height, z-score	0.07 ± 0.23	0.05 ± 0.22	0.08 ± 0.23
Siblings, *n*	0.87 ± 0.78	0.84 ± 0.80	0.90 ± 0.76
No. of family members in the household, *n*	3.07 ± 1.08	2.98 ± 1.05	3.15 ± 1.11
Frequency of attending nursery schools, times/week	5.06 ± 0.36	5.08 ± 0.28	5.03 ± 0.42
Respiratory infections in the past 6 months	3.05 ± 1.18	3.29 ± 1.12	2.85 ± 1.21
Vaccine history			
Rotavirus, *n* (%)	58 (64.5)	36 (73.5)	33 (56.9)
Influenza, *n* (%)	66 (61.1)	27 (55.1)	39 (66.1)

*Values represent the mean ± standard deviation*.

The median (interquartile range) intake rates of the test formula were comparable at 84.1% (66.6, 92.9%), and 75.3% (46.3, 95.1%) for the placebo and LF participants, respectively (*p* = 0.265). The numbers of participants who exhibited some symptoms were: total, 96 (95.0%); acute gastrointestinal symptoms, 52 (51.5%); and acute respiratory symptoms, 94 (93.1%).

Regarding the primary endpoints, the prevalence of acute gastrointestinal symptoms was significantly lower in the LF group (22/53 [41.5%]) than in the placebo group (30/48 [62.5%], *p* = 0.046), whereas the prevalence of acute respiratory symptoms was comparable between the groups during intervention period (placebo: 47/48 [97.9%], LF: 47/53 [88.7%], *p* = 0.115) (Table 2, Supplementary Figure 1). Fecal samples were collected in only 11 children with diarrhea, revealing norovirus in 2 children and *Escherichia coli* O111 (verotoxin negative) in 1 child.

**Table 2:** Acute symptoms observed during the intervention period.

	**Placebo (*n* = 48)**	**LF (*n* = 53)**	**RD (95% CI)**	***p***
**GASTROINTESTINAL SYMPTOM**				
Prevalence, *n* (%)	30 (62.5)	22 (41.5)	0.21 (0.019, 0.401)	**0.046**
Total days	1 (0, 3)	0 (0, 2.5)		0.151
Duration, days/episode	1 (1, 2)	2 (1.38, 3)		0.060
Medication, *n* (%)	12 (40)	13 (59.1)		0.262
**RESPIRATORY SYMPTOM**				
Prevalence, *n* (%)	47 (97.9)	47 (88.7)	0.092 (−0.002, 0.187)	0.115
Total days	15 (6.25, 22.75)	9 (3.5, 18.5)		**0.030**
Duration, days/episode	5 (2.75, 6.33)	4 (2.33, 5.5)		0.194
Medication, *n* (%)	38 (80.9)	37 (78.7)		1.000

*RD, risk difference; CI, confidence interval*.

*Values represent the number (%) or the median (interquartile range)*.

*Prevalence is defined as the number of participants who experienced more than one symptom*.

*Total days is defined as the cumulative number of symptomatic days*.

*Duration is defined as the mean of consecutive days per episode*.

*Risk difference is calculated by subtracting the prevalence in the LF group from the prevalence in the placebo group*.

For the secondary endpoints, the total number of days of acute respiratory symptoms was significantly lower in the LF group (9.0) than in the placebo group (15.0, *p* = 0.030), while the total number of days of gastrointestinal symptoms and the duration of acute respiratory and gastrointestinal symptoms were similar between the groups (Table 2, Supplementary Figure 2). The rate of medication use was also similar and no significant differences were observed between the groups.

In the post-intervention period, the prevalence rate and the total number of days of acute respiratory symptoms were significantly lower in the LF group than in the placebo group (*p* = 0.028 and *p* = 0.010, respectively), while there was no significant difference in acute gastrointestinal symptoms (Table 3, Supplementary Figures 3, 4).

**Table 3:** Acute symptoms observed in the post-intervention period.

	**Placebo (*n* = 48)**	**LF (*n* = 53)**	**RD (95% CI)**	***p***
**GASTROINTESTINAL SYMPTOM**				
Prevalence, *n* (%)	4 (8.3)	3 (5.7)	0.027 (−0.073, 0.127)	0.706
Total days	0 (0, 0)	0 (0, 0)		0.583
Duration, days/episode	1 (1, 1.75)	1 (1, 1)		0.386
Medication, *n* (%)	0 (0)	1 (33.3)		0.429
**RESPIRATORY SYMPTOM**				
Prevalence, *n* (%)	25 (52.1)	16 (30.2)	0.219 (0.031, 0.407)	**0.028**
Total days	1 (0, 6)	0 (0, 1.5)		**0.010**
Duration, days/episode	5 (2, 8)	2 (1.25, 4.75)		0.177
Medication, *n* (%)	9 (36.0)	9 (56.3)		0.334

*RD, risk difference; CI, confidence interval*.

*Values represent the number (%) or the median (interquartile range)*.

*Prevalence is defined as the number of participants who experienced more than one symptom*.

*Total days are defined as the cumulative number of symptomatic days*.

*Duration is defined as the mean of consecutive days per episode*.

*Risk difference is calculated by subtracting the prevalence in the LF group from the prevalence in the placebo group*.

Regarding the medication, there was no difference in medication between the two groups during the intervention and post-intervention period (Tables 2, 3). Antitussive/expectorant, bronchodilator, anti-allergy/anti-histamine, probiotics and antibiotics were used frequently (Supplementary Tables 1, 2).

The duration of fatigue as acute respiratory symptom was significantly shorter in the LF group than in the placebo group. No significant differences were observed for other acute gastrointestinal and respiratory symptoms during the intervention and post-intervention period between the two groups (Tables 4–7).

**Table 4:** Acute gastrointestinal symptoms observed during the intervention period.

	**Placebo (*n* = 30)**	**LF (*n* = 22)**	***p***
**DIARRHEA**			
Prevalence, *n* (%)	26 (86.7)	19 (86.4)	1.000
Diarrhea days	1.5 (1, 3.25)	2 (1, 5.25)	0.393
Duration, days/episode	1 (1, 2)	1.8 (1, 3)	0.417
**VOMITING**			
Prevalence, *n* (%)	15 (50.0)	12 (54.5)	0.785
Vomiting days	0.5 (0, 1)	1 (0, 1)	0.778
Duration, days/episode	1 (1, 1.33)	1 (1, 1.75)	0.867
**FATIGUE**			
Prevalence, *n* (%)	11 (36.7)	11 (50.0)	0.401
Fatigue days	0 (0, 1)	0.5 (0, 1.25)	0.406
Duration, days/episode	1 (1, 1.5)	1 (1, 2)	0.949

*Values represent the number (%) or the median (interquartile range)*.

*Prevalence is defined as the number of participants who experienced more than one symptom*.

*Symptom days are defined as the cumulative number of symptomatic days*.

*Duration is defined as the mean of consecutive days per episode*.

**Table 5:** *Acute respiratory symptoms observed during the intervention period*.

	**Placebo(*n* = 47)**	**LF (*n* = 47)**	***p***
**FEVER**			
Prevalence, *n* (%)	30 (63.8)	29 (61.7)	1.000
Fever days	2 (0, 4)	1 (0, 3)	0.270
Duration, days/episode	2 (1.33, 3)	1.67 (1, 2)	0.216
**NASAL SECRETION/CONGESTION**			
Prevalence, *n* (%)	47 (100)	46 (97.9)	1.000
Nasal secretion/congestion days	12 (4, 21)	10 (4, 18)	0.342
Duration, days/episode	4.25 (2, 6)	4 (2.5, 5.75)	0.942
**COUGH/SPUTUM**			
Prevalence, *n* (%)	45 (95.7)	38 (80.9)	0.050
Cough/sputum days	9 (3, 15)	5 (2, 13)	0.243
Duration, days/episode	4 (2.1, 6)	4 (2, 5.13)	0.956
**FATIGUE**			
Prevalence, *n* (%)	28 (59.6)	27 (57.4)	1.000
Fatigue days	2 (0, 6)	1 (0, 3)	0.197
Duration, days/episode	2.5 (1.54, 4)	1.67 (1, 2)	**0.013**
**OTHERS**			
Prevalence, *n* (%)	4 (8.5)	6 (12.8)	0.740
Others, days	0 (0, 0)	0 (0, 0)	0.534
Duration, days/episode	1 (1, 2.5)	1 (1, 1)	0.610

*Values represent the number (%) or the median (interquartile range)*.

*Prevalence is defined as the number of participants who experienced more than one symptom*.

*Symptom days is defined as the cumulative number of symptomatic days*.

*Duration is defined as the mean of consecutive days per episode*.

*Otitis media and eye discharge associated with other respiratory symptoms*.

**Table 6:** Acute gastrointestinal symptoms observed in the post-intervention period.

	**Placebo (*n* = 4)**	**LF (*n* = 3)**	***p***
**DIARRHEA**			
Prevalence, *n* (%)	1 (25.0)	0 (0)	1.000
Diarrhea, days	0 (0, 0.75)	0 (0, 0)	0.629
Duration, days/episode	1 (1, 1)	– (–,–)	–
**VOMITING**			
Prevalence, *n* (%)	3 (75.0)	3 (100)	1.000
Vomiting, days	1 (0.25, 1)	1 (1, 1)	0.629
Duration, days/episode	1 (1, 1)	1 (1, 1)	1.000
**FATIGUE**			
Prevalence, *n* (%)	2 (50.0)	1 (33.3)	1.000
Fatigue, days	0.5 (0, 1)	0 (0, –)	0.857
Duration, days/episode	1 (1, 1)	1 (1, 1)	1.000

*Values represent the number (%) or the median (interquartile range)*.

*Prevalence is defined as the number of participants who experienced more than one symptom*.

*Symptom days are defined as the cumulative number of symptomatic days*.

*Duration is defined as the mean of consecutive days per episode*.

**Table 7:** Acute respiratory symptoms observed in the post-intervention period.

	**Placebo (*n* = 25)**	**LF (*n* = 16)**	***P***
**FEVER**			
Prevalence, *n* (%)	7 (28.0)	3 (18.8)	0.712
Fever, days	0 (0, 1)	0 (0, 0)	0.682
Duration, days/episode	1 (1, 2)	1.5 (1, –)	1.000
**NASAL SECRETION/CONGESTION**			
Prevalence, *n* (%)	24 (96.0)	16 (100)	1.000
Nasal secretion/congestion, days	6 (2, 8)	2 (1.25, 4.75)	0.101
Duration, days/episode	5 (1.63, 7.75)	2 (1, 4.75)	0.113
**COUGH/SPUTUM**			
Prevalence, *n* (%)	19 (76.0)	11 (68.8)	0.723
Cough/sputum, days	2 (0.5, 6)	1.5 (0, 4.75)	0.361
Duration, days/episode	4 (2, 5)	3 (1, 5)	0.525
**FATIGUE**			
Prevalence, *n* (%)	9 (36.0)	5 (31.3)	1.000
Fatigue, days	0 (0, 1)	0 (0, 1)	0.741
Duration, days/episode	1 (1, 3)	1 (1, 2.5)	0.898
**OTHERS**			
Prevalence, *n* (%)	0 (0)	2 (12.5)	0.146
Others, days	0 (0, 0)	0 (0, 0)	0.517
Duration, days/episode	– (–, –)	1.5 (1, –)	–

*Prevalence is defined as the number of participants who experienced more than one symptom*.

*Symptom days is defined as the cumulative number of symptomatic days*.

*Duration is defined as the mean of consecutive days per episode*.

*Otitis media and eye discharge associated with other respiratory symptoms*.

Thirteen participants in the placebo group and 11 in the LF group experienced adverse events during the intervention period (*p* = 0.357) and 2 and 1, respectively, in the post-intervention period (*p* = 0.587). Major adverse events included eye discharge, asthma, and eczema. No adverse drug reactions were found.

## Discussion

We herein described the first study to investigate the effects of an LF-fortified growing-up formula on acute gastrointestinal and respiratory symptoms in children aged 12–32 months. Our results indicated that an LF-fortified formula can reduce the prevalence of acute gastrointestinal symptoms and the total number of days of respiratory symptoms in children > 1 year of age.

Gastroenteritis is mainly viral or bacterial. Worldwide, rotavirus is considered the most common viral cause of severe acute gastroenteritis, accounting for 453,000 deaths and over 2 million hospitalizations among children under the age of 5 years (17). Although reductions in infection rates have been reported among the 98 countries who introduced rotavirus vaccinations based on recommendations (18), the pathogen remains a leading cause of acute gastroenteritis (19, 20). Norovirus is as the second most frequent cause of severe childhood gastroenteritis. However, it is responsible for the majority of gastroenteritis outbreaks worldwide since no vaccines are currently available (21). Improved methods for preventing such viral infections are important for the health of children worldwide.

LF exerts antiviral effects on rotavirus and norovirus through the inhibition of viral attachment and replication in animals (22, 23). Moreover, oral administration of LF induced interferon (IFN) α/β in the small intestine of mice (24). Therefore, the ingestion of LF may provide protection against acute gastrointestinal illnesses via such mechanisms. In human studies, daily intake of LF-containing yogurt or tablets ameliorated the severity of rotavirus-based gastroenteritis in children (25). LF administration also reduced the duration and severity of diarrhea due to pathogens including norovirus (26). However, such studies mainly showed the alleviation of symptoms. The present report demonstrates that oral LF can reduce the incidence of acute gastrointestinal symptoms in children aged <1 year. Considering the lack of treatments available for gastroenteritis caused by norovirus or rotavirus, daily intake of LF-fortified formula may be a promising alternative to prevent gastroenteritis.

The common cold and influenza are the most common upper respiratory tract infections. In adults, consumption of LF-containing tablets reduced common cold-like symptoms (27), and LF ingestion with milk immunoglobulins decreased the incidence of colds (28). LF was also reported to display antiviral properties against the influenza A virus *in vitro* (29, 30). In an animal model, LF supplementation decreased lung inflammation in mice due to influenza infection (31). No human studies on the impact of LF on influenza have been reported to date. This study was the first to reveal the suppressive effects of LF on respiratory symptoms such as colds in children aged <1 year, although no significant effect on influenza was observed (Supplementary Table 3).

In this study, the prevalence and total number of days of acute respiratory symptoms were significantly lower in the LF group than in the placebo group, even during the post-intervention period. Ishikado et al. (32) reported that the enhanced productivity of IFN-α, an activating factor for NK cells, was not observed 3 weeks after LF ingestion ended. Therefore, the consumption of LF might have increased and sustained natural killer cell cytotoxicity for several weeks after the intervention period and contributed to the suppression of acute respiratory symptoms during the 2-week post-intervention period.

Some studies in adults have reported various beneficial effects of LF at 200 mg/day such as protective effect against infectious gastroenteritis (33), improvement of iron metabolism (34), and acne control (35). Considering the difference in body weight between adults and children, 40–50 mg per day of LF was estimated as an appropriate dose for the participants in this study. Chen et al. have reported that a relatively low dose of LF (35.8 mg/day) suppressed respiratory-related and diarrhea-related illness in infants aged 4–6 months (11), which seems to support this study. Currently, LF is widely fortified in several formula powders, with doses varying from 30.8 to 607.5 mg per day, but a high dose of LF could not be necessarily required to achieve its beneficial effects.

This study had some limitations. First, the data regarding acute respiratory and gastrointestinal symptoms were collected from self-reporting diaries by the parents, which have resulted in a mingling of physician's diagnoses and subjective interpretations of symptoms as determined by the parents. However, in order to minimize the subjective bias, health workers periodically contacted parents and carefully collected information regarding each symptom, with the principal investigator comprehensively judging each symptom as an acute illness or something else (11, 15). Secondly, the pathogens of acute gastrointestinal and respiratory symptoms were not identified. The pathogens of acute respiratory illnesses other than influenza are rarely identified in Japan. The causative pathogens in the most samples were unknown.

In spite of the above limitations, this is the first randomized, double-blind, placebo-controlled trial to investigate the effects of an LF-supplemented growing-up formula on acute respiratory and gastrointestinal symptoms in children aged 12–32 months. This investigation demonstrated that LF ingestion could decrease the prevalence of acute gastrointestinal symptoms and lower the total number of days of acute respiratory symptoms in children aged 12–32 months. It provides important evidence on the beneficial effects of LF for healthy child development.

## Data Availability Statement

The datasets analyzed in this article are not publicly available because participants of this study did not agree for individual data to be shared publicly. Requests to access the datasets should be directed to Noriko Motoki, nmotoki@shinshu-u.ac.jp.

## Ethics Statement

The studies involving human participants were reviewed and approved by the Institutional Review Board of Shinshu university school of medicine. Written informed consent to participate in this study was provided by the participants' legal guardian/next of kin.

## Author Contributions

NM, KY, FA, and TN designed the study. NM, MMiz, TT, MMiy, and MT contributed to data collection. NM, SK, and HO performed statistical analysis and wrote the manuscript. TT, KY, FA, and TN provided critical feedback on the manuscript. All authors reviewed and approved the manuscript.

## Conflict of Interest

MMiy, SK, HO, MT, KY, and FA were employed by Morinaga Milk Industry, Co., Ltd. The remaining authors declare that the research was conducted in the absence of any commercial or financial relationships that could be construed as a potential conflict of interest.
