# Supplementary material for: Effects of Lactoferrin-Fortified Formula on Acute Gastrointestinal Symptoms in Children Aged 12–32 Months: A Randomized, Double-Blind, Placebo-Controlled Trial
Source: Front Pediatr. 2020 May 19;8:233. doi: 10.3389/fped.2020.00233 (PMC7249745; doi:10.3389/fped.2020.00233)
Supplement: Supplementary file 1 [file Data_Sheet_1.docx]

Supplementary Material

Supplementary Table 1.

## Medication types used during the intervention period

|  | Placebo  (n=48) | LF  (n=53) |
| --- | --- | --- |
| Antitussive/ Expectorant, n | 34 | 33 |
| Bronchodilator, n | 18 | 16 |
| Anti-allergy/ Anti-histamine, n | 22 | 20 |
| Probiotics, n | 20 | 19 |
| Antidiarrheal, n | 0 | 1 |
| Antiemetic, n | 0 | 3 |
| Antibiotics, n | 22 | 21 |
| Influenza antiviral drugs, n | 7 | 10 |
| Antipyretics, n | 12 | 9 |
| Others, n | 6 | 2 |

Supplementary Table 2.

## Medication types used in the post-intervention period

|  | Placebo  (n=48) | LF  (n=53) |
| --- | --- | --- |
| Antitussive/ Expectorant, n | 7 | 7 |
| Bronchodilator, n | 3 | 2 |
| Anti-allergy/ Anti-histamine, n | 6 | 4 |
| Probiotics, n | 1 | 3 |
| Antidiarrheal, n | 0 | 0 |
| Antiemetic, n | 0 | 0 |
| Antibiotics, n | 3 | 4 |
| Influenza antiviral drugs, n | 1 | 1 |
| Antipyretics, n | 2 | 0 |
| Others, n | 3 | 1 |

Supplementary Table 3.

## Influenza diagnosed during the intervention period

|  | Placebo  (n=48) | LF  (n=53) | RD (95% CI) | p |
| --- | --- | --- | --- | --- |
| **Influenza** |  |  |  |  |
| Prevalence, n (%) | 7 (14.6) | 10 (18.9) | -0.043 (-0.188, 0.102) | 0.605 |
| Total days | 0 (0, 0) | 0 (0, 0) |  | 0.569 |
| Duration, days/episode | 7 (6, 7) | 6 (5.75, 7.25) |  | 0.887 |
| Medication, n (%) | 7 (100) | 10 (100) |  | - |

Abbreviations: RD, risk difference; CI, confidence interval.

Values represent the number (%) or the median (interquartile range).

Prevalence represents the number of participants diagnosed with influenza..

Total days is defined as the cumulative number of symptomatic days.

Duration is defined as the mean of consecutive days per episodes.

Risk difference is calculated by subtracting the prevalence in the LF group from the prevalence in the placebo group.


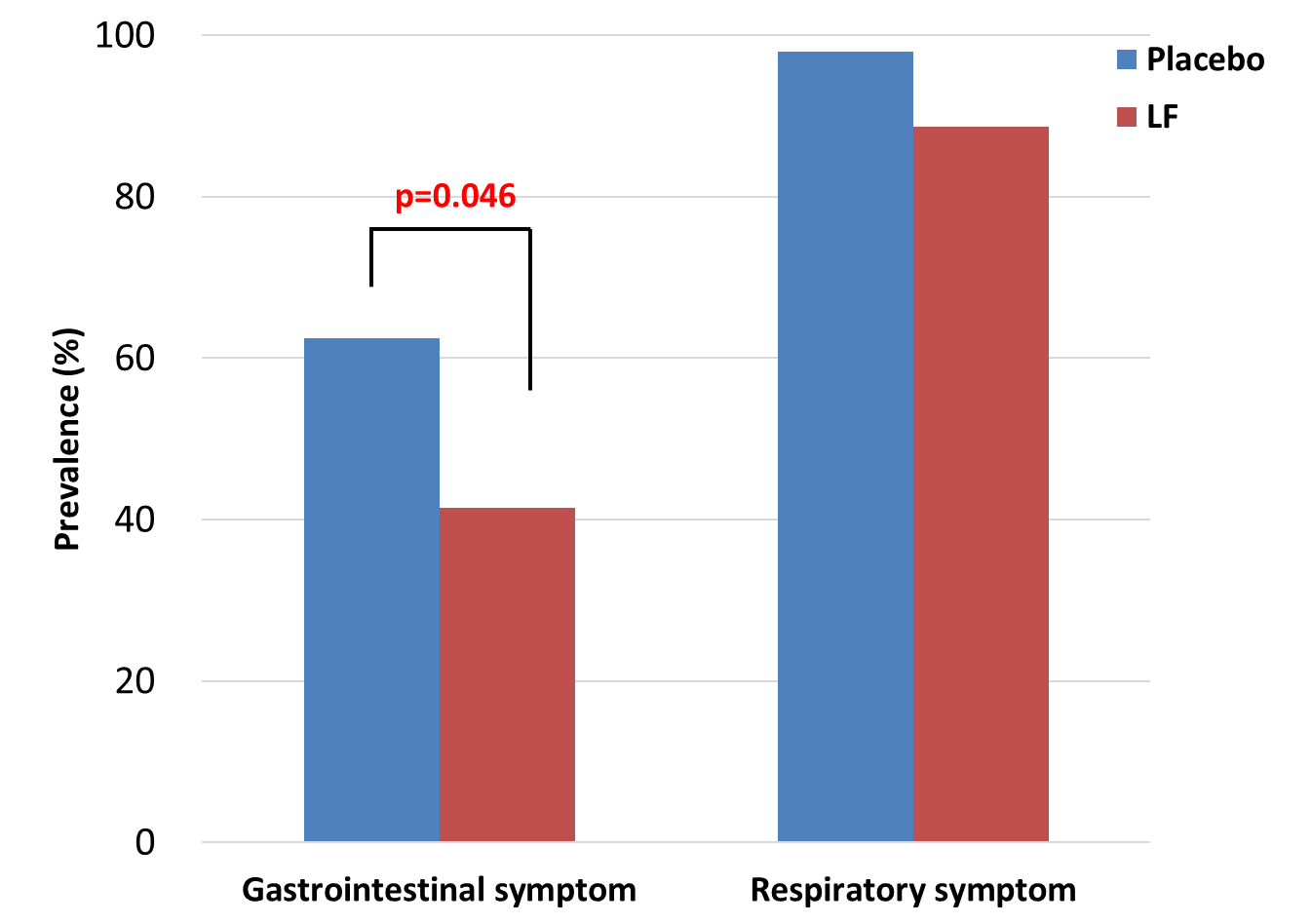


## Supplementary Figure 1.

## Prevalence of acute gastrointestinal and respiratory symptom during the intervention period


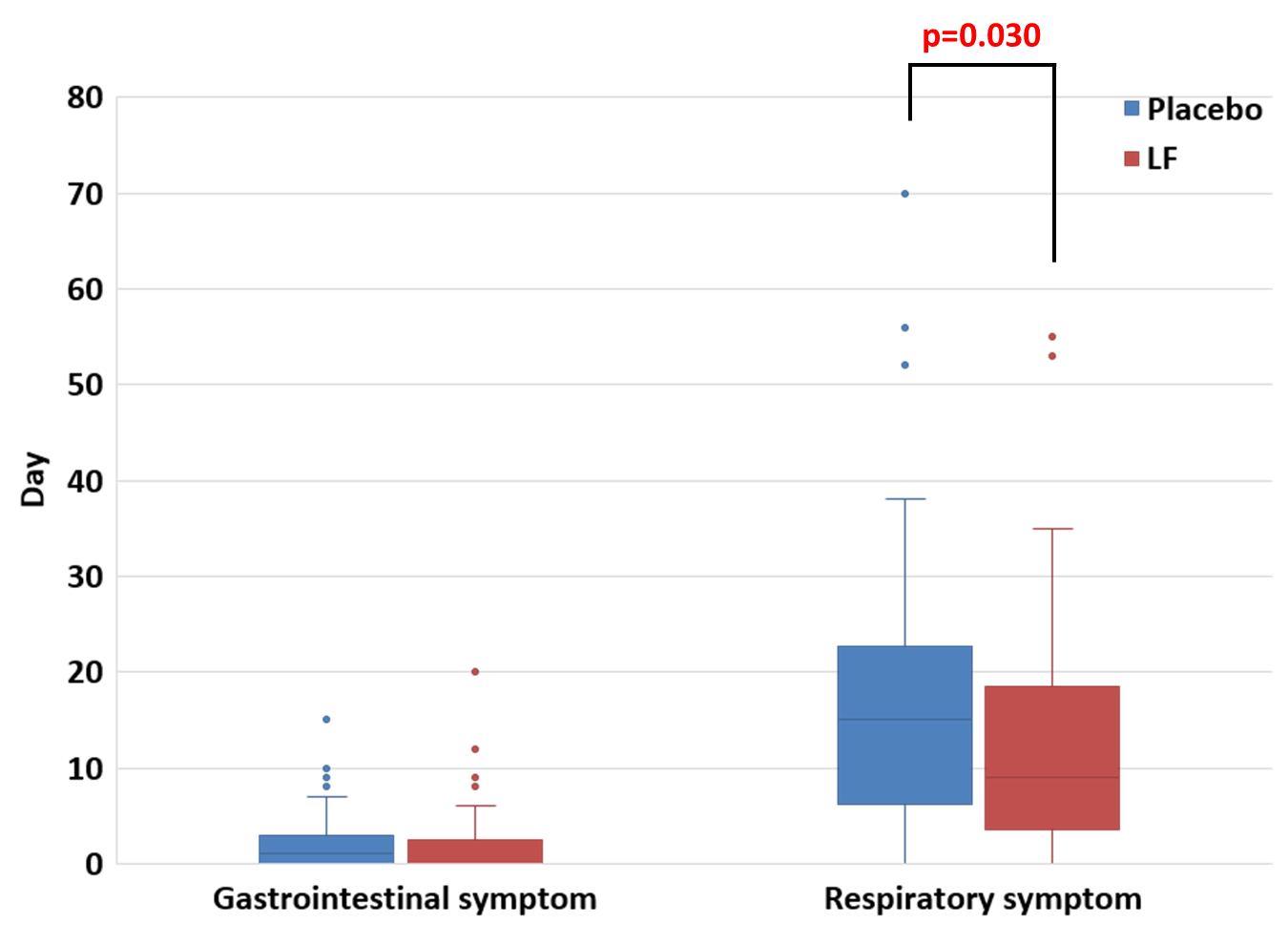


**Supplementary Figure 2.**

**Total number of days of acute gastrointestinal and respiratory symptom during the intervention period**

##
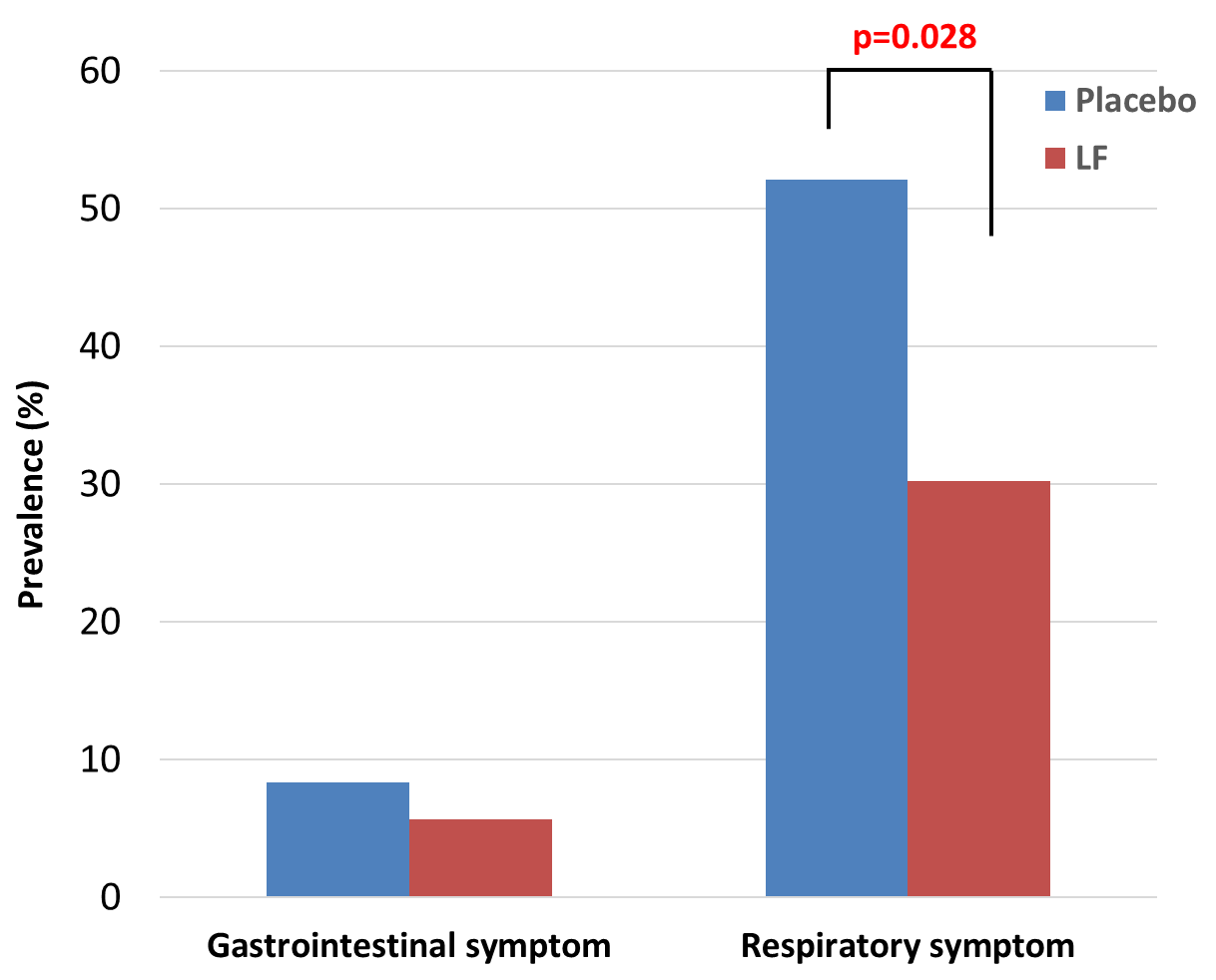


## Supplementary Figure 3.

## Prevalence of acute gastrointestinal and respiratory symptom in the post-intervention period

**
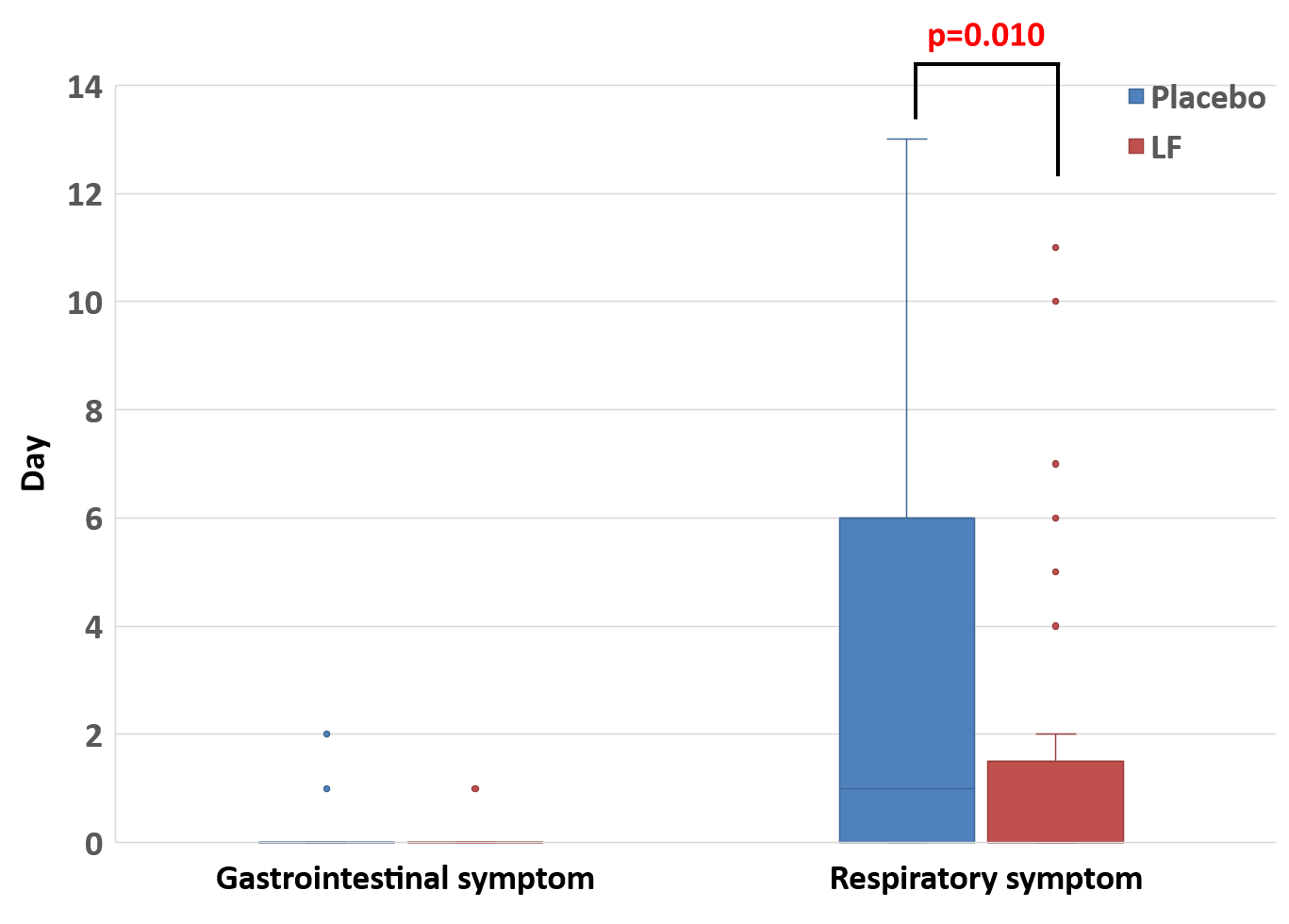
**

**Supplementary Figure 4.**

**Total number of days of acute gastrointestinal and respiratory symptom in the post-intervention period**
